# Status and risk factors of unintentional injuries among Chinese undergraduates: a cross-sectional study

**DOI:** 10.1186/1471-2458-11-531

**Published:** 2011-07-05

**Authors:** Hongying Shi, Xinjun Yang, Chenping Huang, Zumu Zhou, Qiang Zhou, Maoping Chu

**Affiliations:** 1 Department of Preventive Medicine, School of Environmental Science and Public Health, Wenzhou Medical College, Wenzhou 325035, China; 2 Wenzhou Center for Disease Control and Prevention, Wenzhou 325027, China; 3 Department of Psychology, School of Environmental Science and Public Health, Wenzhou Medical College, Wenzhou 325035, China; 4 The First Affiliated Hospital of Wenzhou Medical College, No.2, Fu Xue Road, Wenzhou 325000, China

## Abstract

**Background:**

Injuries affect all age groups but have a particular impact on young people. To evaluate the incidence of non-fatal, unintentional, injuries among undergraduates in Wenzhou, China, assess the burden caused by these injuries, and explore the associated risk factors for unintentional injuries among these undergraduates, we conducted a college-based cross-sectional study.

**Methods:**

Participants were selected by a multi-stage random sampling method, and 2,287 students were asked whether they had had an injury in the last 12 months; the location, cause, and consequences of the event. The questionnaire included demographic and socioeconomic characteristics, lifestyle habits, and the scale of type A behaviour pattern (TABP). Multivariate logistic regression models were used; crude odds ratios (ORs), adjusted ORs and their 95% confidence intervals (CIs) were estimated, with students having no injuries as the reference group.

**Results:**

The incidence of injuries among undergraduates in Wenzhou was 18.71 injuries per 100 person-years (95%CI: 17.12~20.31 injuries per 100 person-years). Falls were the leading cause of injury, followed by traffic injuries, and animal/insect bites. Male students were more likely to be injured than female students. Risk factors associated with unintentional injuries among undergraduates were: students majoring in non-medicine (adjusted OR: 1.53; 95% CI: 1.19-1.96); type A behaviour pattern (adjusted OR: 2.99; 95% CI: 1.45-6.14); liking sports (adjusted OR: 1.86; 95% CI: 1.41-2.45).

**Conclusions:**

Injuries have become a public health problem among undergraduates. Falls were the major cause of non-fatal injury. Therefore, individuals, families, schools and governments should promptly adopt preventive measures aimed at preventing and controlling morbidity due to non-fatal injury, especially among students identified to be at high-risk; such as male students with type A behaviour pattern who like sports.

## Background

Injuries are a growing public health problem, and affect all age groups, but have a particular impact on young people[1,2]. In particular, road traffic injuries are the leading cause of death for those aged between 15 and 29 years[1]. In China, injuries account for more than 10% of all deaths. More than 30% of all potentially productive years of life lost are due to premature mortality. Traffic-related injuries, suicide, drowning, and falls account for 79% of all injury-related deaths [3]. All the data surveyed were focused on fatal injuries, not on the prevalence or incidence of non-fatal injuries. Fatal injuries is only the tip of the 'injury iceberg'[4]. The exclusive report on injury prevention in China from the Ministry of Health showed that the annual rate of injuries for all age groups in China was between 16.1 and 21.9 per 100 population [5], but the rate of injuries among youth was about 50%[6]. Compared with other age groups, injuries among youth, especially among undergraduates, would cause much more direct or indirect economic loss and even more problems to families, to countries, and to our society[7,8].

In the past several decades, many investigations and research studies have been conducted on injuries among young people all over the world [9-17]. Several studies on non-fatal injuries showed that the annual rate of injury ranged from 10 to 50 per 100 students among middle-school and high-school students [9,16,18,19]. However, among undergraduates, non-fatal injury has received little or no attention. One study showed that individuals aged 18-22 had the highest rates of pedestrian injury among any age group in the United States [20]. Another study showed that the incidence of needle stick injuries in medical school was 59% [21]. But the very few studies that have been done among undergraduates are limited to only one or two types of injuries [20-22]; and do not reveal the overall incidence of all injuries among college students, nor do they reflect the overall resulting economic and emotional burdens to society, especially in the context of Chinese colleges.

Previous studies have showed some evidence for an association between health and psychological factors of individuals such as personality and behaviour pattern, especially type A behaviour pattern [23,24]. Type A behaviour pattern (TABP), characterized by time urgency, impatience, alertness, aggressiveness and hostility, was originally developed in relation to coronary heart disease in 1960 [25]. Subsequent epidemiologic studies have examined the relationship between the type A behaviour pattern and road traffic accidents in the United States, Great Britain, France, Nepal, and other countries [26-28]. In China, some researchers also demonstrated the previous findings about the association between TABP and heart diseases, and one study showed that the relationship between type A behaviour pattern and risk driving behaviour, indicating that drivers with type A behaviour pattern were more likely to have aggressive driving attitudes and aggressive driving behaviours [29], but to our knowledge, little evidence was shown on the association between TABP and injuries especially among undergraduates.

Wenzhou, lying on the Southeastern coast of China, is one of the major cities in Zhejiang Province, with a long history, mild climate, and prosperous economy. According to data from the Wenzhou Municipal Bureau of Statistics, there are over 7.5 million of people in the city, one third of whom migrated from other locations, making Wenzhou the largest city in Zhejiang Province. The gross domestic product (GDP) was RMB252,734,000,000 in 2009, and per capita GDP reached over $5000.00. The educational system also developed very rapidly, accepting many undergraduates from all over the world in recent years. There are now 6 colleges (three of them are traditional and the main colleges included in this study) with about 80,000 college students(three quarters of them are in these three main colleges) who come from all parts of the country [30].

In order to describe the current status for non-fatal unintentional injuries among undergraduates in Wenzhou, China, we conducted a college-based cross-sectional study. We aimed to answer the following questions: (1) What is the incidence of nonfatal injuries among the undergraduates in Wenzhou City, China? (2) What is the burden caused by these injuries? (3) Who are more likely to be injured among the undergraduates?

## Methods

### Study subjects

A cross-sectional study was conducted in three main colleges in Wenzhou, China. We used a multi-stage probability sampling method to obtain a representative sample. We used school and grade as stratum, and then selected classes by cluster sampling in each stratum. In total, 2,350 students (aged 17 to 23) were selected from 82 classes and were all surveyed, 2,287 of whom made valid replies, yielding a response rate of 97.3%. The major of students included Medicine, Education, Mathematics, Business, and Management Science.

### Study design

A college-based cross-sectional study was conducted from January to March, 2009. We used self-administered questionnaires (see additional file 1: A transcript of the questionnaire) to survey these randomly selected undergraduates. The questionnaire solicited socio-demographic information and experience of injuries during the preceding 12 months. For each reported event, the student was asked to specify the location where it happened, activities at the time of injuries, the cause, and the consequences of the event.

Additionally, the scale of type A behaviour pattern (TABP) revised by the Chinese National Collaborative Study Group for TABP & coronary heart disease [31] in China was used for all participants, to explore the relationship between type A behaviour pattern and injury. This scale is a revised version of some foreign scales such as the scale of Jenkins Activity Survey (JAS), and has been commonly used in China since the 1980s. It has a good reliability (Cronbach's α coefficient of reliability is 0.78 in this study), and there are 60 items in the scale of TABP, including three dimensions: TH (time hurry), CH (competitive hostility), and L (lie). If the score for the L dimension is higher than 7, the questionnaire is invalid and should be deleted in the final data analysis. The higher the total score on the scale, the more competitive, the more hostile, and the closer to the type A behaviour pattern; otherwise, the lower the total score, the closer to type B behaviour pattern. According to the mean(= 27) and standard deviation(= 8) of the total score of the scale, we divided the students into five groups, similar to previous researchers: A(≥ 36), mA (28~35), M (27), mB (19~26), and B(≤ 18) [31], that is, if the total score on the scale is larger than the mean + standard deviation (SD), then the student will be grouped into type A, if the total score on the scale is less than mean-SD, then the student is grouped into type B, which was a little different from the standard in some other countries [26].

The questionnaire was pre-tested with 60 students from two classes prior to the formal survey. Eight investigators were college students majoring in preventive medicine who received formal training before the survey. During the survey, participants were not allowed to discuss the survey material and they were requested to check for any missed questions before handing in the questionnaire.

In order to avoid selection and drop-out bias, we surveyed the students during the period before the final examination so that almost all students were present. If students were absent at the time of the survey, the class monitor was instructed to give the questionnaires to them upon their return to college, and the investigator would subsequently retrieve the completed survey.

This research study was done in compliance with the Helsinki Declaration, and was reviewed and approved by the Wenzhou Medical College, School of Environmental Science and Public Health. Principals of selected schools signed written consent forms. Students' verbal consent was obtained before the study. Assurance was given that all questionnaire information about the students would be confidential and only used for research.

### Operational injury definition

A reportable injury in this study was defined as an injury meeting at least one of the following criteria [16]: (1) an injury for which the student received medical treatment at the school nurse's office, or received medical care from a doctor at a hospital or a private medical office, (2) an injury for which the student received first aid from his/her schoolmates, teachers, or parents, or (3) an injury that was not treated but caused the student to miss a half day or more of school or regular activities.

For analyses of injuries by cause, we used the tenth Revision of the International Classification of Diseases (ICD-10) and grouped the codes into categories, such as traffic injury, fall, cut, burn, animal/insect bite, poisoning, drowning, choking, and others. The focus of this study was unintentional non-fatal injuries; therefore, child abuse, suicide, and homicide cases were excluded. Unintentional injuries were defined as any injuries that were not deliberately caused by the student or another person [32].

### Data analysis

Analyses were performed with the use of SAS software (version 9.1, SAS Institute) or SPSS statistics package (version 14.0). Considering that there are repeated injuries for some students, besides prevalence, injury incidence was calculated and compared by student gender, age group, family characteristics, including total number of children in the family, marital status, parents' education, and family income. All statistical analyses were conducted by 2-tailed tests with a level of significance set at 0.05.

Logistic regression models were used to control for potential confounding factors, and crude odds ratios and adjusted odds ratios (OR) and 95% confidence intervals (CIs) for OR were calculated. In all regression models, the outcome variable was whether the student had an injury in the past year (if yes, then y = 1; otherwise, y = 0). And in this study, the independent variables included individual level determinants (gender, age, major, behaviour pattern, sports), and family socioeconomic determinants (family annual per-capita income, the number of siblings), we used different independent variables in different models for controlling specific factors in every model.

## Results

We sent out 2,350 questionnaires, and received 2,287 valid replies. 62 students were excluded because of invalid or missing replies; only one student was excluded because of reporting intentional injury. Among the respondents, 954 (41.7%) were male students, and 1,333 (58.3%) were female students. The mean age was 19.5 years, and the standard deviation 1.1 years. A total of 1,088 students were medical students, and 1,199 students were not.

### Injury incidence

Among the 2,287 respondents, a total of 428 injuries requiring medical attention were reported for 320 students during the year preceding the interview, leading to an incidence of 18.71 injuries per 100 person-years. The corresponding 95% confidence interval (95%CI) for this incidence was 17.12~20.31 injuries per 100 person-years. However, the percentage of students injured during the year was 13.99% (320/2287), with 95% confidence interval (95%CI) at 12.57%~15.41%. Among this group of 320 students, 23.44% had multiple episodes of injury during the preceding 12 months.

Table 1 presents the incidence of injuries by gender and cause of injuries. As recorded, falls were the leading cause of injury, followed by traffic injuries, bites, and burns. There are some differences between male and female students; the three leading causes for male students are falls, traffic injuries, and bites respectively; but for female students the three leading causes are falls, traffic injuries, and burns, respectively. The incidence for falls of male students was 15.41 injuries per 100 person-years, which was significantly higher than that of female students (8.78 injuries per 100 person-years); and for burns, female students have significantly higher incidence than males (table 1). In addition, for both males and females, sports were the most common activities associated with injury.

**Table 1:** Incidence of unintentional injuries by gender and cause among undergraduates in Wenzhou, China

Cause of injury	Male (n = 954)			Female (n = 1333)			Total (n = 2287)		
	
	*n*	Injuries per 100 person-years (95%CI)		*n*	Injuries per 100 person-years (95%CI)		*n*	Injuries per 100 person-years	
Falls*	147	15.41	(13.12~17.70)	117	8.78	(7.26~10.29)	264	11.54	
Traffic injuries	25	2.62	(1.61~3.63)	29	2.18	(1.39~2.96)	54	2.36	
Animal/insect bites	15	1.57	(0.78~2.36)	14	1.05	(0.50~1.60)	29	1.27	
Cuts	10	1.05	(0.40~1.69)	8	0.60	(0.19~1.01)	18	0.79	
Burns*	4	0.42	(0.01~0.83)	24	1.80	(1.09~2.51)	28	1.22	
Poisoning	7	0.73	(0.19~1.27)	13	0.98	(0.45~1.50)	20	0.87	
Drowning/Choking	1	0.10	(-0.10~0.31)	1	0.08	(-0.07~0.22)	2	0.09	
Others	2	0.21	(-0.08~0.50)	11	0.83	(0.34~1.31)	13	0.57	

Total	211	22.12	(19.48~24.75)	217	16.28	(14.30~18.26)	428	18.71	

* *P *< 0.05.

**Figure 1:**
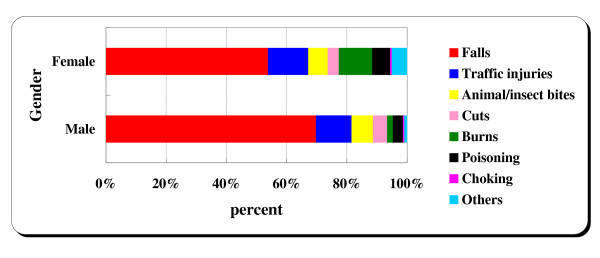
**Proportions of non-fatal unintentional injuries by cause for both male and female undergraduates**. Falls = fall on same level from slipping, tripping, collision, and fall from stairs/ladder; Traffic injuries = injured in collision with pedal cycle, motor vehicle, car, bus; Animal/insect bites = bitten by rats, dogs, and nonvenomous insect; Cuts = cut by knife, sword, sharp glass; Burns = fire and burn/scald; Poisoning = drug, alcohol poisoning; Choking = Inhalation and ingestion of food causing obstruction of respiratory tract, accidental drowning; Others = injury from electric current, radiation, product, consumer goods, etc.

Then, we analyzed the proportions of reported medically attended non-fatal injuries by cause for both male and female students. As Figure 1 shows, the proportions of female injuries by cause were very similar to that of male injuries. For both male and female students, falls were the most common cause of injury, as depicted in Figure 1.

### Epidemiological characteristics of injuries among undergraduates

**Figure 2:**
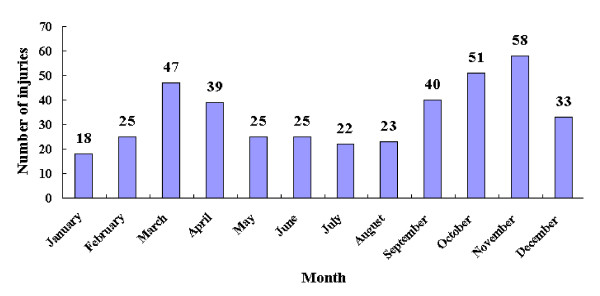
**Time distribution of non-fatal injuries among undergraduates in Wenzhou, Zhejiang, China**. 406 injured cases reported the month injured, and were used to explore the time distribution of injuries in this figure. 22 cases did not report the time injured, and were excluded from this analysis.

We analyzed the time distribution of injuries among college students, and found that there seemed to be two peaks for non-fatal injuries among these students. As Figure 2 shows, one peak was in spring, the other was in autumn.

We found that 66.9% of the 428 injured cases occurred on campus, 12.9% of them on the roads, 10.7% at home, and 9.5% at other locations. All participants in this study came from three different colleges in Wenzhou city. Our survey found that the incidence of injury for students from medical college was 17.5 injuries per 100 person-years (209 injuries/1192 person-years), the incidence for students from vocational college was 15.0 injuries per 100 person-years (32/213), whereas that for students from a comprehensive university was 21.2 injuries per 100 person-years (187/882). Meanwhile, the prevalence for these three colleges was 12.6%, 16.7%, 10.8% respectively, and there was a significant difference among them (χ^2 ^= 9.01, *P *= 0.011).

In addition, among the 1,140 students from rural areas, 207 injuries occurred, resulting in an incidence of 18.2 injuries per 100 person-years; whereas students from urban areas had an incidence of 19.3 injuries per 100 person-years, but the difference of the percentage injured between these two groups(13.2% vs 14.7%) was not significant (χ^2 ^= 1.05, *P *= 0.305).

We analyzed other characteristics of injuries among undergraduates, and found that male students, students majoring in non-medicine, students from a one-child family, students from low-income families, students with type A behaviour, and students liking sports and activities tend to experience the highest prevalence of injuries.

As indicated in table 2, the incidence for male students was 22.1 injuries per 100 person-years (95% CI: 19.5, 24.7), while that for female students was 16.3 injuries per 100 person-years (95% CI: 14.3, 18.3); the difference of percentage of students injured between male and female students was statistically significant (χ^2 ^= 4.08, *P *= 0.043). Medical students had an incidence of 15.9 per 100 person-years (95% CI: 13.7, 18.1), and that for non-medical students was 21.3 (95% CI: 19.0, 23.6), the difference of percentage of students injured between these two groups was also statistically significant. Likewise, students with no sibling had higher injury rates than those with ≥ 1 sibling. The injuries for students from families with an annual per-capita income less then 2,000 Yuan was the highest with an incidence of 28.3 per 100 person-years (95% CI: 22.6, 34.0), however, the difference of percentage of students injured between these five different groups was not significant. The incidence for students with type A behaviour pattern was 52.3 injuries per 100 person-years (95% CI: 37.5, 67.0), which was higher than that of any other type of behaviour pattern, and the percentages of students injured among the difference types of behaviour patterns were also significantly different. Students who liked sports or took part in club activities in college experienced significantly higher rates than those who disliked sports or didn't attend club activities.

**Table 2:** Relationship between demographic and psychological characteristics and injuries among undergraduates in Wenzhou, China

Characteristics	*n*	Number of injuries (injuries per 100 person-years)	Number of persons injured (percentage, %)	**χ**^**2**^	*P*
Gender					
Male	954	211(22.1)	150(15.7)	4.08	0.043
Female	1,333	217(16.3)	170(12.8)		
Major					
Medicine	1,088	173(15.9)	122(11.2)	13.32	< 0.001
Non-medicine	1,199	255(21.3)	198(16.5)		
No. of siblings					
0 (only child)	849	184(21.7)	135(15.9)	4.09	0.043
≥ 1 sibling	1,438	244(17.0)	185(12.9)		
Family income levels*					
< 2,000	240	68(28.3)	41(17.1)	7.15	0.128
2,000~10,000	610	94(15.4)	69(11.3)		
10,000~30,000	658	126(19.1)	103(15.7)		
30,000~50,000	342	59(17.3)	48(14.0)		
> 50,000	437	81(18.5)	59(13.5)		
Behaviour Pattern					
A	44	23(52.3)	12(27.3)	13.36	0.010
mA	462	97(21.0)	77(16.7)		
M	122	28(23.0)	19(15.6)		
mB	1,070	194(18.1)	146(13.6)		
B	589	86(14.6)	66(11.2)		
Sports					
Dislike	843	110(13.1)	83(9.8)	19.07	< 0.001
Like	1,444	318(22.0)	237(16.4)		
Club activities					
Yes	921	141(15.3)	109(11.8)	5.96	0.015
No	1,366	287(21.0)	211(15.4)		

* Annual per-capita income, Yuan

Based on the above univariate and bivariate analyses, and in order to control for other confounding factors, we conducted multivariate logistic regression analyses, and found some factors that may be associated with non-fatal injuries among undergraduates. As shown in table 3, non-medical students experienced significantly higher rates than medical students, the odds ratios (ORs) changed very little even after adjusting for gender or other variables, which indicate students' major may be an influencing factor for non-fatal injuries. Consistent with the results from bivariate analyses, students who like sports are more likely to be injured than those that do not like sports (adjusted OR = 1.86, 95%CI: 1.41~2.45).

**Table 3:** Odds ratios (ORs) with 95%CI for non-fatal Injuries among undergraduates in Wenzhou, China

Characteristic	Crude OR (95%CI)	**Adjusted OR**^**a **^**(95%CI)**	**Adjusted OR**^**b **^**(95%CI)**
**MAJOR**			
Medicine	1	1	1
Non-medicine	1.57(1.23~2.00)	1.53(1.20~1.95)	1.53(1.19~1.96)
**TABP**			
B	1	1	1
mB	1.25(0.92~1.71)	1.23(0.90~1.68)	1.17(0.85~1.60)
M	1.46(0.84~2.54)	1.43(0.82~2.48)	1.44(0.82~2.51)
mA	1.58(1.11~2.26)	1.55(1.08~2.20)	1.47(1.03~2.11)
A	2.97(1.46~6.05)	2.86(1.40~5.84)	2.99(1.45~6.14)
**SPORTS**			
Dislike sports	1	1	1
Like sports	1.80(1.38~2.35)	1.74(1.33~2.29)	1.86(1.41~2.45)
**No. of siblings**			
≥ 1 sibling	1	1	1
0 (only child)	1.28(1.01~1.63)	1.23(0.96~1.57)	1.25(0.98~1.61)

a: Adjusted for gender.

b: Adjusted for gender and all variables in table 3.

After adjusting for gender or other variables, Type A behaviour pattern (TABP) still remained statistically significant in the model. Compared with the students with type B behaviour pattern, students with type A behaviour pattern had a higher risk of injury (adjusted OR = 2.99, 95%CI: 1.45~6.14).

From family level, we found that students who had no siblings were more likely to be injured than other students; however, this difference became non-significant after adjusting for gender or other variables (Table 3).

### Burden caused by injuries

Of all injured students, 3.16% were hospitalized, 56.45% were treated as outpatients, 21.41% were treated by themselves or other persons, and 18.98% were not treated at all. Most (74.94%) were minor injuries, and no disability was found. As for the anatomical location of the injury, 44.1% were wounded at the lower extremity, 19.4% the upper extremity, 12.2% the head and face, and 6.6% were injured at multiple parts.

On average, injuries led to 7.28 days of rest(however, 56.0% of the cases had no rest, 44.0% had a mean of 15.8 days of rest, median = 7 days, the first quartile was 2 days, the third quartile was 16 days), 0.55 days of hospitalization (96.8% of them had no hospitalization, only 3.2% of the cases were hospitalized and had a mean of 15.3 days of hospitalization, median = 7 days, the first quartile was 1.5 days, the third quartile was 21 days), and 0.53 work days off for family members (87.1% of the cases had no work days off for family members, 12.9% had a mean of 6.9 days, median = 2 days, the first quartile was 1 day, the third quartile was 10 days). The modes of the days of rest and hospitalization were both 7 days. Traffic injuries, and falls caused the longest period of both hospitalization and days of rest.

The distribution of the direct costs caused by injuries from treatments was right skewed, these 320 students resulted in the sum of all treatment fees at 115 224.90 yuan (mean = 360.08 yuan); however, 31.5% of the injuries resulted in no treatment fees, and the 5% trimmed mean of direct costs from injuries was 119.52 yuan, with the median of 20.00 yuan, and an interquartile range of 187.50 yuan. The highest costs that were caused by traffic injury amounted to 12,000 yuan.

## Discussion

In this study, the incidence of injuries among undergraduates in Wenzhou city, Zhejiang Province, China, was 18.71 injuries per 100 person-years, which is consistent with the results of previous studies [9,11,19]. This is slightly higher than the results (15.64%) of four communities in Zhejiang province, the same province as our study[33], and also higher than the 6.67% incidence of Shandong Province, China [2]. However, this incidence is lower than the rate among middle-school and high-school students in Guangxi Province, China [16].

Falls are the leading cause of injuries among college students, which is similar to previous studies among other populations[1,2,33]. Falls are the most common cause of injuries for both male and female students. The three leading causes for male students are falls, traffic injuries, and bites; but for female students the three leading causes are falls, traffic injuries, and burns.

The study also showed that the annual injury rate was higher among male students than female students, which is similar to the results among primary and middle school students [2,3,11].

Type A behaviour pattern (TABP), characterized by impatience, time urgency, and hostility, was originally developed in relation to coronary heart disease [34]. In this study, we also found that type A behaviour pattern may be correlated with undergraduate students' injuries. This, possibly, is because type A students were more likely to engage in risky behaviours, such as walking more rapidly, performing activities more quickly, being more impatient, and doing different things at the same time [26,28]. These results indicate that students themselves with type A behaviour pattern should be more careful when performing exercise or other activities; what's more, teachers in colleges and universities should pay more attention to these high risk groups with type A behaviour pattern, when they take measures such as health education, to reduce or prevent injuries among college students.

Medical students experienced lower rates than non-medical students. This is possibly because medical students have more knowledge about health and are more likely to protect themselves after learning medical theories and develop a greater sense of self-preservation-awareness than other students[35,36].

Some researches found that socioeconomic status (SES) was an important risk factor for injuries[37-39]. In this study, we also found that the family economic status may be a risk factor for nonfatal injuries. Students with low family income level are more likely to be injured. Policy makers and investigators should use multiple measures of SES and target family income level or social level in attempting to reduce the rate of unintentional injuries.

We also found that students from different schools have different rates of injuries, which indicates that there may be a hierarchical or clustered structure in the data set [40]; for instance, students from the same school tend to be more alike in some characteristics than individuals chosen at random from the population at large.

Considering that we drew a random sample from the main three colleges in Wenzhou, so the results may be generalizable to all college students in these three main colleges in Wenzhou. There are about 80,000 college students now in Wenzhou; therefore, about 11,000 students (based on the percentage of students injured of 13.99%) can be assumed to have suffered from a total of between 14,000 and 16,000 injuries (based on 95% CI of the incidence: 17.11~20.31 injuries per 100 person-years) in Wenzhou every year. The direct economic costs attributed to these 11,000 non-fatally injured persons would be about 4,000,000 yuan (calculated by multiplying 11,000 by 360.08 yuan). The indirect costs would, of course, be even greater. However, at present, education focusing on injury prevention and control is still infrequent and fragmented, even in medical schools around the world [41,42].

Some potential limitations of this study have been identified: firstly, although the study reported the incidence by asking for a recount of the past 12 months, there must be some innate recall bias in a cross-sectional study, and we did not include the students who went on educational internship, which may result in underestimating the incidence. The evidence provided by a cross-sectional study is not strong enough to draw any causal inferences [43]. In order to confirm the relationship between factors including type A behaviour pattern and risk of injury, a prospective cohort study should be conducted. In addition, because we found that different clusters (schools) had different injury epidemiological characteristics in the survey, it would be better to use multilevel models or generalized linear mixed models (GLMM) [44,45] to more accurately estimate the relationship between individual (age and gender), and cluster variables (school type), and injury, more accurately.

## Conclusions

In summary, non-fatal injuries have been a public health problem among college students in Wenzhou City, China, and produced many direct and indirect economic costs. Falls are the leading cause of injuries among college students in Wenzhou city, China. Type A behaviour may be a risk factor for injuries, even after controlling for other confounding factors. Thus, individuals, families, schools and governments should all participate in the process of injury prevention and control, focusing on the main causes of injury, reducing the rate of injury and the burdens of injury; thereby improving the health and safety of college students in Wenzhou city.

## List of abbreviations used

CI: Confidence interval; OR: odds ratio; TABP: type A behaviour pattern; SD: standard deviation

## Competing interests

The authors declare that they have no competing interests.

## Authors' contributions

HYS designed the study, conducted the data analysis, and completed the first draft of this article. MPC participated in study design and coordination. XJY and QZ participated in the design of the study, and revised the manuscript. CPH, ZMZ made valuable suggestions on scholarly writing. All authors have read and approved the final manuscript.

## Pre-publication history

The pre-publication history for this paper can be accessed here:

http://www.biomedcentral.com/1471-2458/11/531/prepub

## Supplementary Material

Additional file 1 **A transcript of the questionnaire**. Click here for file
