# Supplementary material for: Status and risk factors of unintentional injuries among Chinese undergraduates: a cross-sectional study
Source: BMC Public Health. 2011 Jul 5;11:531. doi: 10.1186/1471-2458-11-531 (PMC3142514; doi:10.1186/1471-2458-11-531)
Supplement: Additional file 1 — A transcript of the questionnaire. [file 1471-2458-11-531-S1.DOC]

###### Questionnaire for Unintentional Injuries among Undergraduates

Hi, everyone：

Injuries are a growing public health problem in China. We are conducting a survey to gather information, about the patterns of unintentional injuries among university undergraduates; a scientifically clear understanding of the impact of unintentional injuries on university undergraduates will be used for making recommendations to all appropriate officials, on methods for reducing unintentional injuries among this important segment of China’s population. You have been randomly selected to participate in this study. Your participation will contribute to helping prevent injuries not only for your generation of undergraduates, but for future generations as well. Please carefully read and fill-in the questionnaire as accurately as you can remember. The information you provide will be strictly confidential, and will only be used for research purposes.

Thank you for your participation.

―― Department of Preventive Medicine, Wenzhou Medical College

**Instructions: please check“√” the number to indicate which response that best fits your experience.**

College: (1)Wenzhou Medical College (2)Wenzhou University (3)Wenzhou Vocational College

Grade: ________________ Major:__________________ Student id:

**A、basic information**

1. Class：(1)State-run undergraduate (2)Privately-run undergraduate (3)Junior college student □
2. Gender： （1）male （2）female □
3. Birthday：________year ________month _______day □□
4. Ethnic group：（1）han （2）other （_____________） □
5. Height：_________cm □□□
6. Weight：_________kg □□□
7. Your birth place：（1）city （2）rural （3）town □
8. Myopia：（1）yes （2）no □
9. Your rank among brothers and sisters: 1)only child 2)eldest 3)second 4)third or later □
10. Father’s education：（1）illiteracy or primary school（2）junior middle school（3）senior high school （4）junior college （5）college or higher □
11. Mother’s education：（1）illiteracy or primary school（2）junior middle school（3）senior high school （4）junior college （5）college or higher □
12. Father’s vocation：（1）worker （2）farmer （3）official （4）teacher （5）medical （6）businessman （7）military （8）jobless （9）other（_______________） □
13. Mother’s vocation：（1）worker （2）farmer （3）official （4）teacher （5）medical （6）businessman （7）military （8）jobless （9）other（_______________） □
14. Family annual per capita income（yuan）：

（1）<2000（2）2000~10000（3）10000～30000 （4）30000～50000 （5）>50000 □

1. How many persons in your family（who live together）：________persons □
2. Are you in a single parent family：（1）yes （2）no □

If yes, who do you live with? 1)father 2)mother 3)grandma or grandpa 4)others □

1. How long do you usually study everyday?

（1）<4h （2）4~8h （3）8~12h （4）>12h □

1. Study mark：（1）excellent （2）average （3）below average □
2. Are you satisfied with your major? (1) Yes, satisfied. (2) general. (3) No, unsatisfied. □
3. Degree of study pressure：（1）little （2）middle （3）large □
4. Do you smoke?（≥1 stick/day, more than half a year）： （1）yes （2）no □
5. Do you drink? （≥1 time/day，more than 3 months）： （1）yes （2）no □
6. Do you like sports？ （1）yes （2）no 　　　　　 □
7. Do you have a part-time job while studying at college？ （1）yes （2）no □
8. Do you join a club activity？ （1）yes （2）no □
9. Character：（1）introvert （2）extrovert □

**B、Injury Status：**

| **Instructions：**injury means meeting at least one of the following criteria:  (1) an injury for which the student received medical treatment at the school nurse’s office, or received medical care from a doctor at a hospital or a private medical office,  (2) an injury for which the student received first aid from his/her schoolmates, teachers, or parents,  (3) an injury that was not treated but caused the student to miss a half day or more of school or regular activities. |
| --- |

1. Did you have injuries during the preceding 12 months: ①yes，how many times____， ②no

〔*if yes, continue to next item；if no, jump to next part*.〕

1. Time when you were injured：_________year _­­­­­­­­­­­­­­­­_______month
2. Injury type：①traffic injury ②fall ③drowning ④accidental poisoning ⑤accidental choke ⑥burn ⑦mechanical wound ⑧electrical injury ⑨Animal/insect bites ⑩earthquake or typhoon ⑾suicide ⑿homicide ⒀other
3. Place injured：①home ②campus ③road ④amusement building ⑤pond ⑥other
4. Cause of injuries**:____________________（see notes after table 1. ）
5. Injured part：①head or face ②neck ③chest or belly ④waist or back ⑤upper extremity ⑥lower extremity ⑦multiple part ⑧other______
6. Treatment after injury：①hospitalization ②outpatient treatment ③treated by teachers or classmates ④no treatment
7. Treatment fee：__________________ yuan
8. Where did your treatment fee come from：①public expense ②medical insurance ③cooperative medical care ④self-paid ⑤other
9. Weather when injured：①fine ②cloudy ③rainy ④snow ⑤windy ⑥foggy ⑦other
10. How many days did you stay in hospital after injury：_________days
11. How many days did you rest after injury：_________days
12. How many days did your injury delay your family’s work：_________days
13. Degree of injury：①slight（outpatient or rest less than one day） ②moderate（hospitalization between one and ten days） ③severe（hospitalization more than ten days） ④disabled
14. Who caused the injury? ①myself ②other people ③other things

**Table 1 injury information**

| **item** | **The first time** | **The second time** | **The third time** |
| --- | --- | --- | --- |
| 28. time injured |  |  |  |
| 29. injury type |  |  |  |
| 30. place injured |  |  |  |
| 31. cause of injuries * |  |  |  |
| 32. injured part |  |  |  |
| 33. treatment after injury |  |  |  |
| 34. treatment total fee |  |  |  |
| 35. where did your treatment fee come from |  |  |  |
| 36. weather when injured |  |  |  |
| 37. how many days did you stay in hospital after injury |  |  |  |
| 38. how many days did you rest after injury |  |  |  |
| 39. how many days did your injury delay your family’s work |  |  |  |
| 40. degree of injury |  |  |  |
| 41. who caused the injury |  |  |  |

****cause of injuries：**

Traffic injuries: bike, motorcycle, tricycle, car, bus, truck, wet floor, other reason

Falls: slip, stumble, crowded, poor lighting, unsafe stairs, bad shoes, mood, others

Drowning: swimming, slip, poor facilities, other

Poisoning: food, pesticide, drug, gas, other

Choke: bone, fish bone, disease, other

Burns: boiling water, hot oil, fire, chemicals, food, other

Mechanical wound: cut, crash, sport, crush, other

Electrical injury: high-voltage line, poor insulation of the electric wires, electric shock, other

Animal/insect bites: dog, cat, bee, snake, mice, other

Suicide: pressure, economy, family, emotion, depression, other

**C、Scale of type A behavior pattern (TABP)**

**Instructions:** Please answer the following questions based on your personal interpretation of yourself. If the item correctly describes you, please check “yes,” otherwise, check “no.” PLEASE ANSWER ALL QUESTIONS. There is no right or wrong answer. Please answer quickly, without thinking too much about every item. Don’t think what the right answer should be, just answer accurately, based on how you perceive yourself.

1、I always try to persuade others to agree with me. （1）yes （2）no

2、I walk quickly, even though I have no important things to do. （1）yes （2）no

3、I often feel that I have many things to do, and feel pressured. （1）yes （2）no

4、If I decide to do something, it will be difficult for others to change my idea. （1）yes （2）no

5、Some people and some things frequently annoy me. （1）yes （2）no

6、If I need to wait for a long time to buy some important things, I would rather not buy.

（1）yes （2）no

7、If I can’t arrange time for work, I just find time to do it temporarily. （1）yes （2）no

8、I am never late for work or appointments. （1）yes （2）no

9、If someone interrupts me when I am doing something, no matter whether he(she) is deliberately interrupting, I am always very annoyed. （1）yes （2）no

10、I always hate to see such persons who always speak slowly and act unhurriedly. （1）yes （2）no

11、I often feel very busy because there are so many things to do. （1）yes （2）no

12、I work well with others; but I always want to do some important parts alone. （1）yes （2）no

13、Sometimes I want to swear at people. （1）yes （2）no

14、I always do something slowly, think many times and can’t make a decision. （1）yes （2）no

15、If someone jumps the queue when I line up for tickets, I will blame them or admonish them.（1）yes （2）no

16、I think I am a light-hearted person and live a life of ease. （1）yes （2）no

17、Sometimes I think what I am worrying about is much more than what I should worry about.（1）yes （2）no

18、Whatever I do, even though I do it worse than others, it doesn’t matter to me. （1）yes （2）no

19、Whatever I do, I never hurry in doing it. It’s useless to worry about it, and if I take it easy, it will not delay anything. （1）yes （2）no

20、I never want to do something according to what I think. （1）yes （2）no

21、I am always very nervous about things everyday. （1）yes （2）no

22、Even when I stroll around the park, admire the beauty of flowers, watch fishes, I always finish early and then wait for others. （1）yes （2）no

23、I often can’t excuse others’ shortcomings. （1）yes （2）no

24、I like everyone that I know. （1）yes （2）no

25、When I hear someone state wrong views, I always want to correct them. （1）yes （2）no

26、Whatever I do, I do it quicker than others. （1）yes （2）no

27、When someone is rude to me, I will also be impolite to them. （1）yes （2）no

28、I think I can do everything well. （1）yes （2）no

29、I am always hurrying to express my opinion, even interrupting others when chatting.

（1）yes （2）no

30、People think I am a steady, calm, and patient person. （1）yes （2）no

31、I think that there are very few persons who are worth believing in and admiring among the persons I know. （1）yes （2）no

32、I have many ideas and plans to the future, and always want to accomplish it as soon as possible. （1）yes （2）no

33、Sometimes I gossip about others. （1）yes （2）no

34、I eat quickly in spite having plenty of time. （1）yes （2）no

35、If I hear someone speak or report badly, I will feel anxiously, and think it should be better done by me. （1）yes （2）no

36、Even though others bully me, I will not care about it. （1）yes （2）no

37、Sometimes I would delay what should be done today for tomorrow. （1）yes （2）no

38、People think I am an efficient person. （1）yes （2）no

39、If someone picks at me or my work, it will destroy my enthusiasm easily. （1）yes （2）no

40、I often feel that it’s late, but it is in fact early. （1）yes （2）no

41、I think I am a very sensitive person. （1）yes （2）no

42、I usually do things in a hurry, and try to do more things with the least time. （1）yes （2）no

43、If I make a mistake, I will be active in acknowledging it whether it is big or not. （1）yes （2）no

44、When taking the bus, I often think it’s driven too slowly. （1）yes （2）no

45、For everything others do, I will not do it, even though I know they cannot do it well. （1）yes （2）no

46、I am often anxious that I didn’t finish my work, but time is past. （1）yes （2）no

47、If I am responsible for many things, it will be much better than now. （1）yes （2）no

48、Sometimes I think about unspeakable ideas. （1）yes （2）no

49、Even though my leader has low ability, I can also obey and cooperate. （1）yes （2）no

50、When there is something that needs waiting for, I will burn with anxiety and lack patience. （1）yes （2）no

51、I often think I have no enough ability, and want to give it up when having difficulty in doing something. （1）yes （2）no

52、I watch TV, and see films everyday, otherwise, I will be uncomfortable. （1）yes （2）no

53、If other person requests me that I do something, I will never delay in doing it as long as I promised. （1）yes （2）no

54、People think that I am patient, and do not hurry in doing anything. （1）yes （2）no

55、If I make an appointment with somebody, I will rarely be late, and if they’re late, I will be angry. （1）yes （2）no

56、Occasionally, I tell lies. （1）yes （2）no

57、Many things can be shared by many people, but I like to do it alone. （1）yes （2）no

58、I think that others understand what I say very slowly and that some cannot understand me. （1）yes （2）no

59、I am easily angered. （1）yes （2）no

60、I usually see the shortcomings of others and ignore their advantages. （1）yes （2）no

**Please check whether you have filled in all items, thank you.**

If you agree, we wish to keep in touch with you.

name________________ phone_______________

**Investigator______________**

**Checker______________**

**Survey date：______year _____month _____day**
